# Supplementary material for: Artificial nanovesicles for dsRNA delivery in spray‐induced gene silencing for crop protection
Source: Plant Biotechnol J. 2023 Jan 18;21(4):854–65. doi: 10.1111/pbi.14001 (PMC10037145; doi:10.1111/pbi.14001)
Supplement: Supplementary file 1 — Figure S1 Genes targeted through SIGS‐applied dsRNAs are down‐regulated in Botrytis cinerea. The expression of the B. cinerea target genes was down‐regulated as measured by RT‐PCR. Figure S2 Treatment with AV‐dsRNA provides prolonged protection against B. cinerea in rose petals. (a) Rose petals were pre‐treated with naked‐ or AV‐Bc‐VDS‐dsRNA, for 1, 3 and 7 days, then inoculated with B. cinerea. Pictures were taken at 3 dpi. (b) The relative lesion sizes were measured with the help of ImageJ software. Error bars indicate the SD. Statistical significance compared to water (Student's t‐test): *P < 0.05. Figure S3 Direct comparison of AV formulation performance with dsRNA performance. Tomato fruits were pre‐treated with naked‐ or AV(DOTAP+PEG)‐Bc‐VDS‐dsRNA, AV(DOTAP)‐Bc‐VDS‐dsRNA, and AV(DODMA)‐Bc‐VDS‐dsRNA, for 1, 5 and 10 days, then inoculated with B. cinerea. Relative lesion sizes were measured with the help of ImageJ software. Error bars indicate the SD of the three biological replicates. Statistical significance compared to naked‐Bc‐VDS (Student's t‐test): *P < 0.05. Figure S4 Treatment with AV‐dsRNAs reduces expression of targeted genes in B. cinerea. From tomato treatments shown in Figure 6, relative gene expression of the three targeted genes in Botrytis, VPS51, DCTN1 and SAC1 was quantified by qPCR using RNA extracted from the infected fruits at 5 dpi. Statistical significance compared to water (Student's t‐test): *P < 0.05; **P < 0.01. Figure S5 AV formulations display no signs of phytotoxicity on Arabidopsis plants. Five‐week‐old Arabidopsis plants were treated with a 20 μL suspension on each leaf of either water, DOTAP+PEG‐, DOTAP‐ or DODMA‐AVs. AV treatments were performed at working concentrations or at two times the working concentration. Working concentration is determined by the amount of lipid needed to encapsulate dsRNA at a concentration of 20 ng/μL. Table S1 Size distribution of unloaded and dsRNA‐loaded AV formulations. Size distributions and zeta [file PBI-21-854-s001.pdf]

Supplementary Figures

Supplemental Figure 1

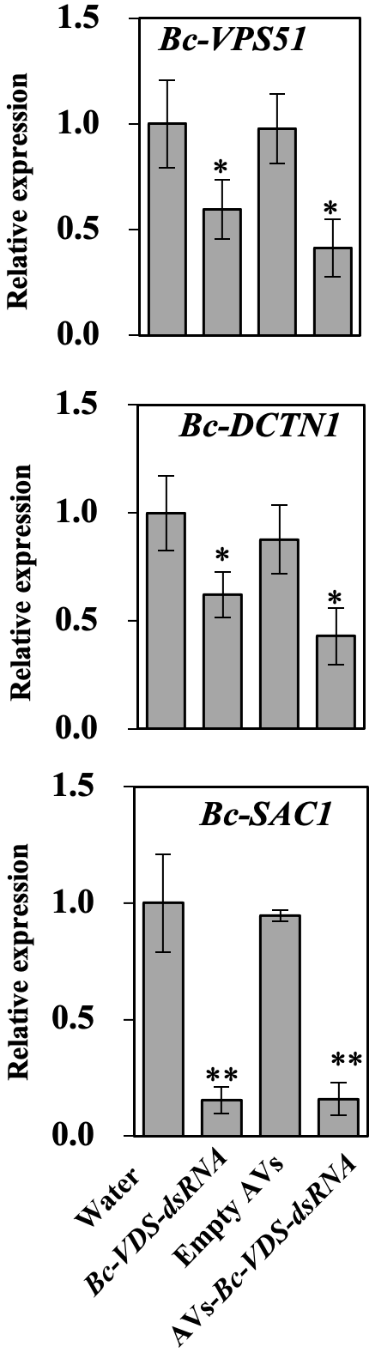

Supplementary Figure 1. Genes targeted through SIGS-applied dsRNAs are downregulated in *Botrytis cinerea.* The expression of the *B. cinerea* target genes were down regulated as measured by RT-PCR.

Supplemental Figure 2

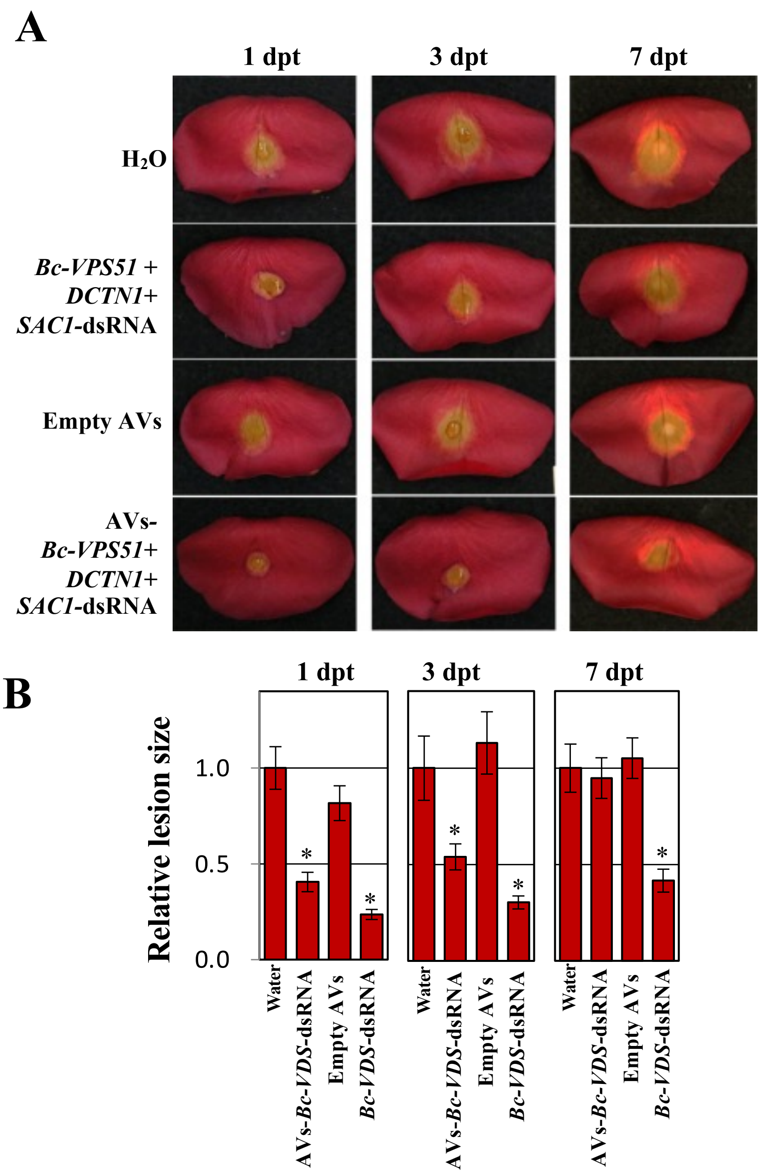

Supplementary Figure 2. Treatment with AV-dsRNA provides prolonged protection against *B. cinerea* in rose petals. (A) Rose petals were pre-treated with naked- or AV-*Bc-VDS*-dsRNA, for 1, 3, and 7 days, then inoculated with *B. cinerea.* Pictures were taken at 3 dpi. **(B)** The relative lesion sizes were measured with the help of ImageJ software. Error bars indicate the SD. Statistical significance compared to water (Student's t-test): *, $P < 0.05$.

Supplemental Figure 3

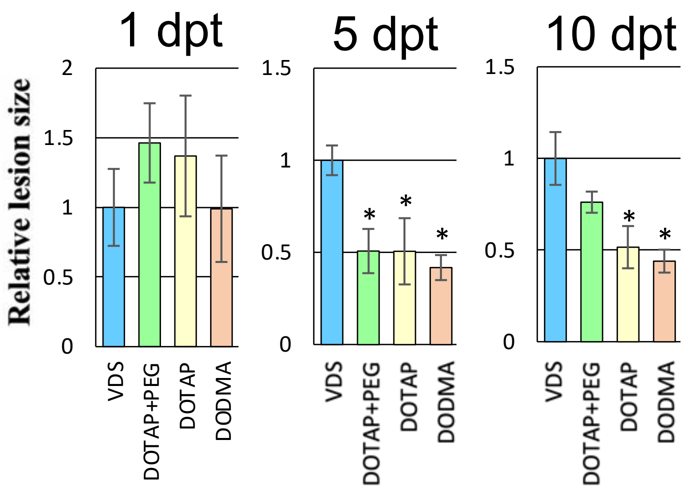

Supplementary Figure 3. Direct comparison of AV formulation performance with dsRNA performance. Tomato fruits were pre-treated with naked- or AV(DOTAP+PEG)-*Bc*-*VDS*-dsRNA, AV(DOTAP)-*Bc*-*VDS*-dsRNA and AV(DODMA)-*Bc*-*VDS*-dsRNA, for 1, 5, and 10 days, then inoculated with *B. cinerea.* Relative lesion sizes were measured with the help of ImageJ software. Error bars indicate the SD of the 3 biological replicates. Statistical significance compared to naked-*Bc*-*VDS* (Student's t-test): *, $P < 0.05$.

Supplemental Figure 4

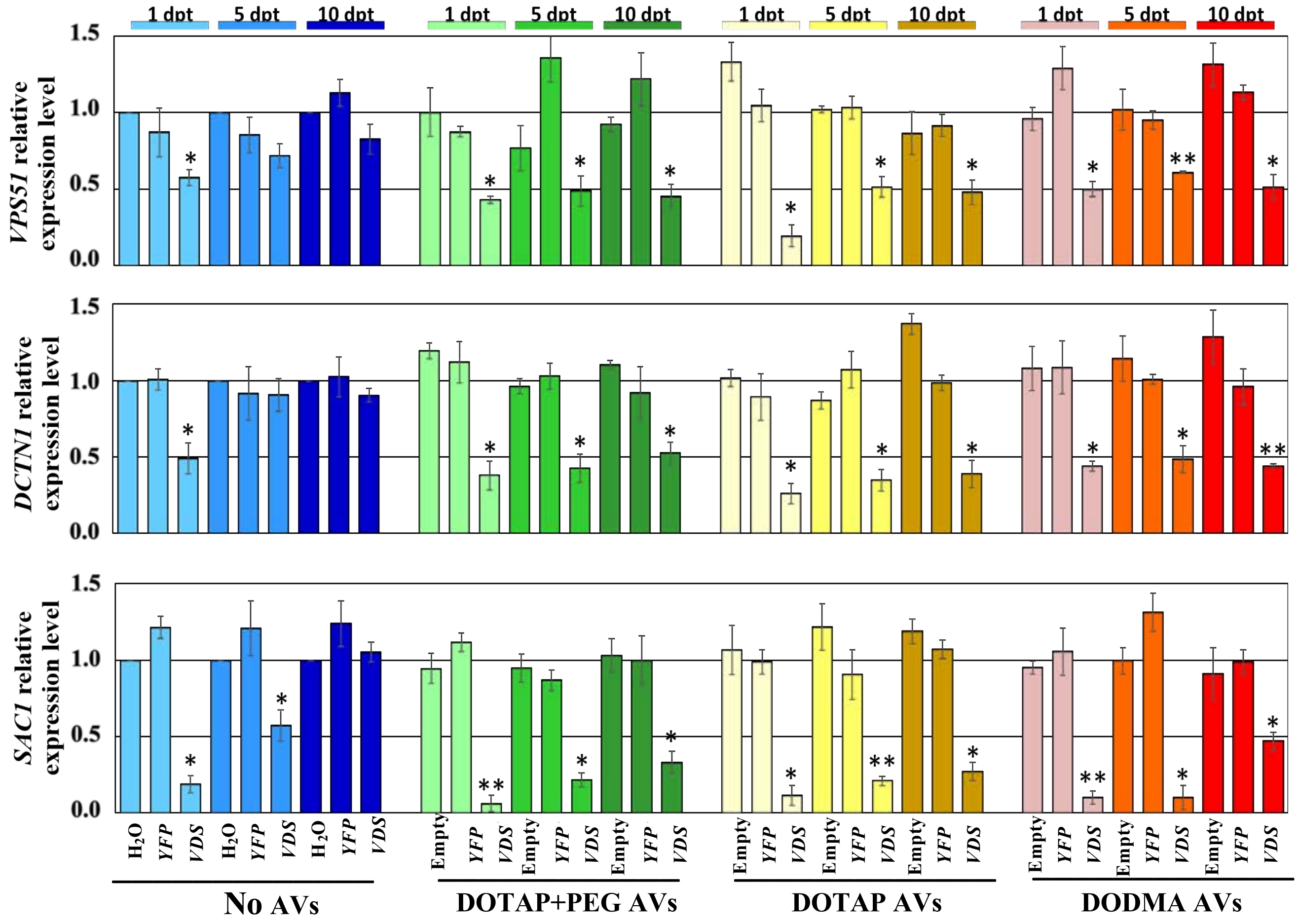

Supplementary Figure 4. Treatment with AV-dsRNAs reduces expression of targeted genes in *B. cinerea*. From tomato treatments shown in Figure 6, relative gene expression of the three targeted genes in *Botrytis*, *VPS51, DCTN1,* and *SAC1* was quantified by qPCR using RNA extracted from the infected fruits at 5 dpi. Statistical significance compared to water (Student's t-test): *, $P < 0.05$; **, $P < 0.01$.

Supplemental Figure 5

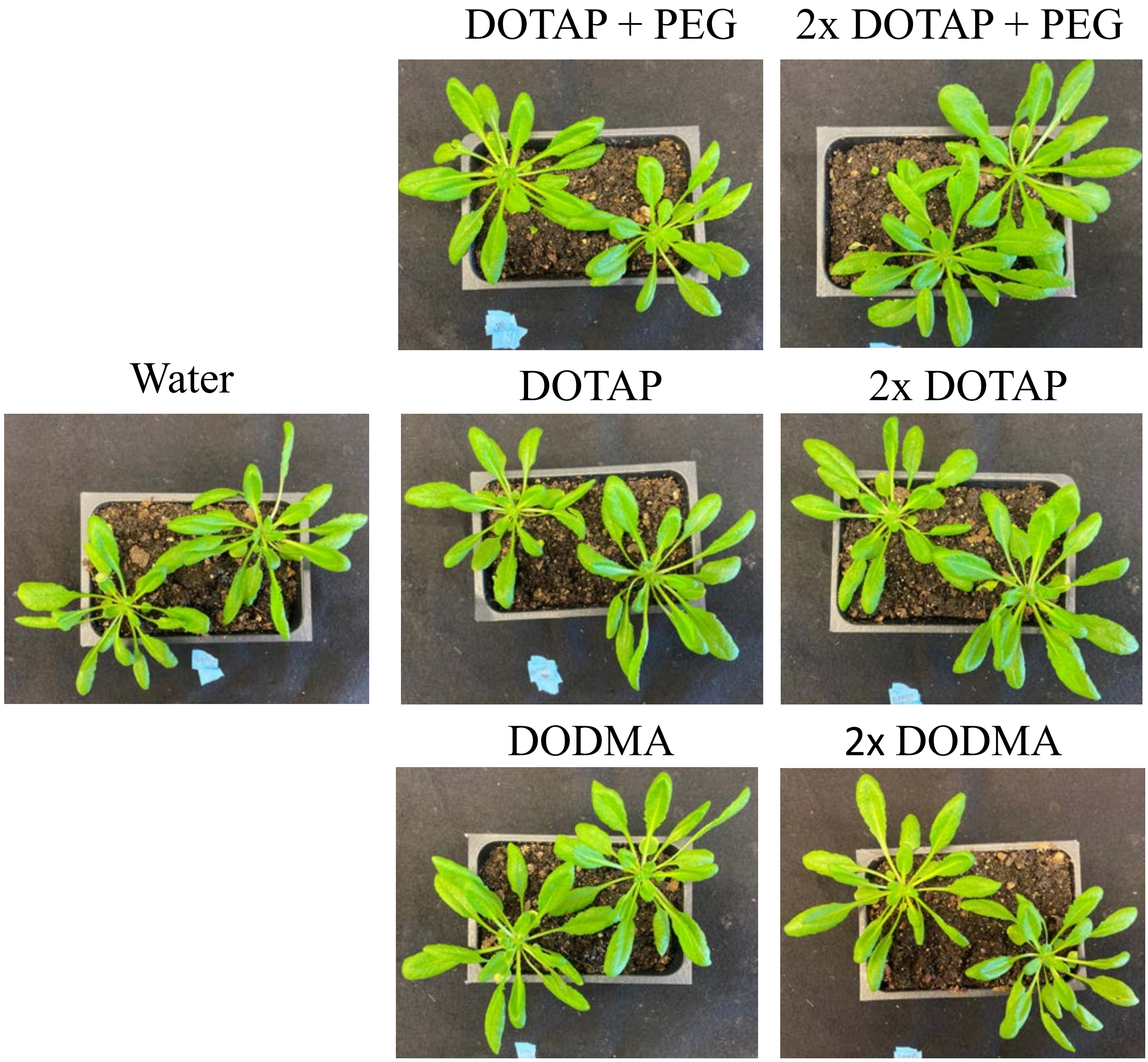

Supplementary Figure 5. AV formulations display no signs of phytotoxicity on *Arabidopsis* plants. 5-week-old *Arabidopsis* plants were treated with a 20 μL suspension on each leaf of either water, DOTAP+PEG-, DOTAP-, or DODMA-AVs. AV treatments were performed at working concentrations or at two times the working concentration. Working concentration is determined by the amount of lipid needed to encapsulate dsRNA at a concentration of 20ng/μL.

Supplementary Tables

Supplemental Table 1: Size distribution of unloaded and dsRNA-loaded AV formulations

Mixing protocol	N/P	Z-avg (d.nm)	PDI	Zeta potential (mV)
Empty DOTAP:Chol:DSPE-PEG2000 (2:1:0.1)	N/A	120.3	0.06	44.32
VDS-dsRNA+DOTAP:Chol:DSPE-PEG2000 (2:1:0.1)	(4:1)	365.3	0.455	47.17
Empty DOTAP:Chol (2:1)	N/A	123.4	0.06	57.59
VDS-dsRNA+DOTAP:Chol (2:1)	(4:1)	259.5	0.368	47.23
Empty DODMA:Chol (2:1)	N/A	298.3	0.337	38.77
VDS-dsRNA+DODMA:Chol (2:1)	(4:1)	393.6	0.385	-9.55

Supplemental Table 2: Primers used in this study

Primer Name	Primer Sequence (5'-3')	Primer Description
YFP-dsRNA-T7-F	TAATACGACTCACTATAGGGAGAATGGTGAGCAAGGGCGAGGA	Template DNA for in vitro YFP-dsRNA synthesis
YFP-dsRNA-T7-R	TAATACGACTCACTATAGGGAGATTACTTGTACAGCTCGTCCATGC	
BcDCL1-dsRNA-T7-F	TAATACGACTCACTATAGGGAGATGCGGAAGAACTTGAAGGTTTGCTACA	Template DNA for In vitro Bc-DCL1/2 synthesis
BcDCL1-dsRNA-T7-R	GCAGCAAATGGCATCCGTCCAGATCTGGTCAACACACCAAG	
BcDCL2-dsRNA-T7-F	CTTGGTGTGTTGACCAGATCTGGACGGATGCCATTTGCTGC	
BcDCL2-dsRNA-T7-R	TAATACGACTCACTATAGGGAGAACTCTTGAGTACTTTCGCCAGCTCAC	
BcVPS51-dsRNA-T7-F	TAATACGACTCACTATAGGGAGATTCGTTCCAGGAGTTACACG	Template DNA for In vitro Bc-VPS51+DCTN1+SAC1 synthesis
BcVPS51-dsRNA-T7-R	CTTTCGACGAGAGCACGAATGATGAGACAAGTGAGAGTCCA	
BcDCTN1-dsRNA-T7-F	TGGACTCTCACTTGTCTCATCATTCGTGCTCTCGTCGAAAG	
BcDCTN1-dsRNA-T7-R	CACTGCACTTTGAACAACGTCCAGCTTACAACTGTGCTCT	
BcSAC1-dsRNA-T7-F	AGAGCACAGTTGTAAGCTGGACGTTGTTCAAAGTGCAGTG	
BcSAC1-dsRNA-T7-R	TAATACGACTCACTATAGGGAGA CCTTCAATGCTGCTGTAGAAG	
BcVPS51-qRT-F	GTTAGGCAAAGTAGGAGAAGG	qRT-PCR BcVPS51, BcDCTN1, and BcSAC1 expression
BcVPS51-qRT-R	CATCCATTCGCCCCAATACC	
BcDCTN1-qRT-F	GCTTCCCTTACCTCGAATTC	
BcDCTN1-qRT-R	CAACCTCTAGTTCTTCCGGTC	
BcSAC1-qRT-F	CGAAAAGTTTCGAGGCATTTG	
BcSAC1-qRT-R	GAACTTAATGGGGTCAAAGCC	
Tomato-tubulin-qRT-F	GATTTGCCCACTAACCTCTCGT	qRT-PCR for relative fungal biomass assays
Tomato-tubulin-qRT-R	ACCTCCTTTGTGCTCATCTTACCC	
Bc-actin-qRT-F	TGCTCCAGAAGCTTTGTTCCAA	
Bc-actin-qRT-R	TCGGAGATACCTGGGTACATAG	
